# Distributed Fading Memory for Stimulus Properties in the Primary Visual Cortex

**DOI:** 10.1371/journal.pbio.1000260

**Published:** 2009-12-22

**Authors:** Danko Nikolić, Stefan Häusler, Wolf Singer, Wolfgang Maass

**Affiliations:** 1 Department of Neurophysiology, Max-Planck-Institute for Brain Research, Frankfurt, Germany; 2 Frankfurt Institute for Advanced Studies (FIAS), Johann Wolfgang Goethe University, Frankfurt, Germany; 3 Institute for Theoretical Computer Science, Graz University of Technology, Graz, Austria; Weill Cornell Medical College, United States of America

## Abstract

It is currently not known how distributed neuronal responses in early visual areas carry stimulus-related information. We made multielectrode recordings from cat primary visual cortex and applied methods from machine learning in order to analyze the temporal evolution of stimulus-related information in the spiking activity of large ensembles of around 100 neurons. We used sequences of up to three different visual stimuli (letters of the alphabet) presented for 100 ms and with intervals of 100 ms or larger. Most of the information about visual stimuli extractable by sophisticated methods of machine learning, i.e., support vector machines with nonlinear kernel functions, was also extractable by simple linear classification such as can be achieved by individual neurons. New stimuli did not erase information about previous stimuli. The responses to the most recent stimulus contained about equal amounts of information about both this and the preceding stimulus. This information was encoded both in the discharge rates (response amplitudes) of the ensemble of neurons and, when using short time constants for integration (e.g., 20 ms), in the precise timing of individual spikes (≤∼20 ms), and persisted for several 100 ms beyond the offset of stimuli. The results indicate that the network from which we recorded is endowed with fading memory and is capable of performing online computations utilizing information about temporally sequential stimuli. This result challenges models assuming frame-by-frame analyses of sequential inputs.

## Abstract 2

The brain has a one-back memory for visual stimuli. Neural responses to an image contain as much information about the current image as it does about another image presented immediately before.

## Author Summary

Researchers usually assume that neuronal responses carry primarily information about the stimulus that evoked these responses. We show here that, when multiple images are shown in a fast sequence, the response to an image contains as much information about the preceding image as about the current one. Importantly, this memory capacity extends only to the most recent stimulus in the sequence. The effect can be explained only partly by adaptation of neuronal responses. These discoveries were made with the help of novel methods for analyzing high-dimensional data obtained by recording the responses of many neurons (e.g., 100) in parallel. The methods enabled us to study the information contents of neural activity as accessible to neurons in the cortex, i.e., by collecting information only over short time intervals. This one-back memory has properties similar to the iconic storage of visual information—which is a detailed image of the visual scene that stays for a short while (<1 s) when we close our eyes. Thus, one-back memory may be the neural foundation of iconic memory. Our results are consistent with recent detailed computer simulations of local cortical networks of neurons (“generic cortical microcircuits”), which suggested that integration of information over time is a fundamental computational operation of these networks.

## Introduction

A number of analysis methods have been designed to investigate how neuronal spiking activity correlates to sensory stimulation or behavior and, as expected, many relations have been found. All of the following variables—firing rates [1]–[4] and the synchronization [4]–[7] and the differential timing of spiking activity [8]–[10]—have been reported to correlate, one way or another, with stimuli and/or with behavior. Most of the efforts to distinguish between relevant and epiphenomenal variables face the problem that they have to rely on correlative rather than causal evidence. In the absence of direct access to the information of causal nature, one way to begin addressing this issue is to complement the highly specialized statistical methods that have been developed to detect specific stimulus-related changes in responses [9],[11]–[18] with analyses that make minimal assumptions about putative codes [19]–[21], and examine how much information can be extracted by a single cortical neuron from the joint responses of its presynaptic neurons and then find out which variables were carrying the relevant information [22],[23]. To adjust to the conditions under which these cortical “readout” neurons are likely to operate, the data must be presented to the readout units in parallel, i.e., the responses of the putative feeding neurons must be recorded simultaneously. Also, analysis and classification of data should correspond to that conducted in real time. Thus, the results should be available with short delays. Hence, the analysis method should be allowed no more time for accumulating evidence than is available for cortical neurons to accomplish their task. To fulfill these requirements, readout neurons need to be simulated on a computer and fed with data obtained in parallel recordings from a large number of cortical neurons, the assumption being that a certain fraction of these neurons provides input to neuronal classification.

The present study was designed to fulfill these premises. Applying multielectrode technology, we recorded simultaneously from a large number of neurons in area 17 of lightly anesthetized cats and evoked responses with brief sequences of different, stationary flashed stimuli (letters of the alphabet). These responses were then fed as inputs to artificial readout neurons simulated on a computer (also referred to as *classifiers*). We first trained the artificial neurons to classify the visual stimuli that have generated the responses, and once the classification had reached criterion, we investigated which of the information-carrying variables were used by these artificial—but neuron-inspired—readout systems. A recent study provided evidence that an artificial readout system can extract stimulus-specific information from the spiking activity of neurons that were sequentially recorded from macaque inferotemporal (IT) cortex [23]. However, in this study, responses of different neurons that were supplied in parallel to the readout system had been obtained by recording sequentially from the different cells. This approach eliminates temporal relations between neuronal responses that are not time locked to the stimulus, but established by internal interactions (referred to also as “noise correlation” [24]). Thus, it was not possible to determine whether stimulus-specific information is encoded by internal adjustment of spike timing, as this information cannot be retrieved unless responses are recorded simultaneously.

In addition to the identification of response variables containing stimulus-specific information, we were particularly interested in determining the duration over which this information was retrievable after the stimuli have disappeared. In case there was evidence for “fading memory” already in primary visual cortex, the data would allow for inferences on the mechanisms underlying perceptual phenomena such as visual persistence and iconic storage [25]–[29]. Moreover, the lack of evidence for fading memory would provide an important constraint for hypotheses on processing modes implemented in primary visual cortex. Computer simulations of sparsely connected recurrent circuits of neuron-like processing units [30]–[33] as well as theoretical analyses of related circuits with simpler (linear) processing units [34],[35] have shown that systems with fading memory exhibit powerful computational capabilities because they permit integration of stimulus information over time. Negative evidence of fading memory would render such models unlikely and support more classical views that emphasize sequential step-by-step processing of single frames resulting from a pipelined organization of the circuits [36],[37].

In order to distinguish between processing with or without fading memory, we presented sequences of different stimuli and investigated whether information about preceding stimuli was erased by the presentation of subsequent stimuli or whether it persisted. The latter case would provide strong evidence for the existence of fading memory, whereas the former would be supportive of models favoring frame-by-frame analysis of successive stimuli.

## Results

### Methods

We used silicon-based multielectrodes (Michigan probes, see Materials and Methods) to obtain highly parallel recordings from up to 124 randomly selected neurons in the primary visual cortex (area 17) of lightly anesthetized cats. These neurons, most of which had overlapping receptive fields (RFs), were activated by presenting stationary flashed (100 ms) uppercase letters (A, B, C, D, and E). The letters were always shown with maximal contrast and were either presented singly, one per trial, or in a sequence of two or three (e.g., ABC; and a 100-ms blank interval between letters in most experiments) (see Table S1 for experiments made on each cat). The times at which letters were presented are indicated in all graphs by gray rectangles. Each stimulation condition was presented 50 to 300 times in a randomized order.

**Figure 1:**
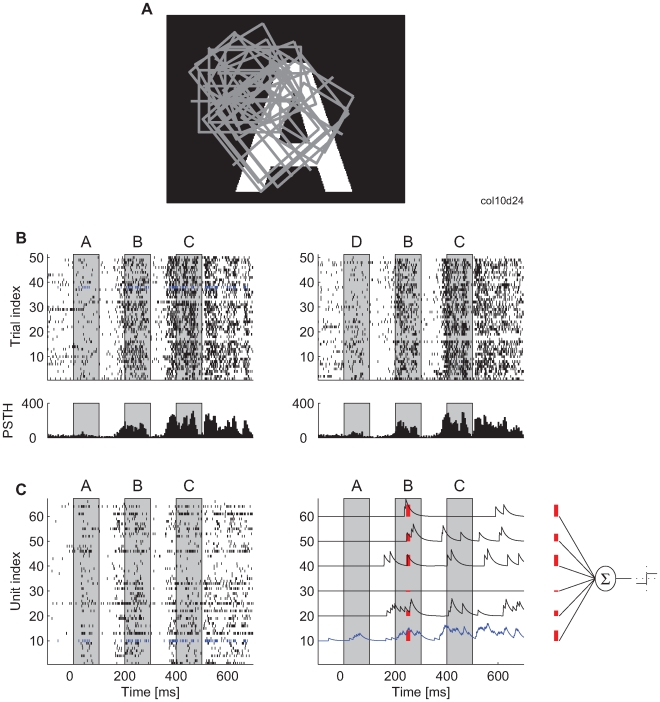
Experimental setup and illustration of the analysis method. (A) An example of a visual stimulus in relation to the constellation of receptive fields (rectangles) from one Michigan probe. (B) Upper part: spike times recorded from one neuron across 50 stimulus presentations and for two stimulus sequences (ABC and DBC). In this and in all other figures, the gray boxes indicate the periods during which the letter stimuli were visible on the screen. Lower part: peristimulus time histogram (PSTH) for the responses of this neuron (5-ms bin size). (C) Left: spike trains obtained simultaneously from 66 neurons in one stimulation trial. Blue: the neuron for which all 50 trials are shown in (B). Right: for the classification analysis, each spike train is convolved with an exponential kernel (i.e., low-pass filtered). The spike trains for only six example neurons are shown. Red: example values of the convolved trace that are used as inputs to the classifier (far right).

To emulate classification processes realized with leaky integrate-and-fire (I&F) readout neurons (but without reset or a refractory period), the time stamps of discharge sequences were first convolved with an exponentially decaying kernel (if not specified otherwise, time constant, *τ* = 20 ms) that mimicked the time course of excitatory postsynaptic potentials (EPSPs) [38],[39] (see Materials and Methods). The continuous convolved signal was fed to the classifier system, as illustrated in Figure 1C. If not specified otherwise, the classification was made by a single I&F readout whose activation, at a time point, *t*, was computed as the weighted sum of the input signals at that time. The classifier “fired” (i.e., detected the stimulus) if its activation crossed a threshold. The optimal weights and the firing thresholds of the classifiers were found (learned) by applying support vector machines (SVM) with linear kernels. All reported results were obtained from test datasets derived from trials that were not part of the training datasets, only the latter being used to adjust the input weights for the readout neurons (see Materials and Methods for a 10-fold cross-validation scheme). In a few additional analyses (reported later in “Superposition of Information about Different Stimuli”), we used also SVMs with polynomial and radial basis function kernels to investigate whether classification performance improved with these more sophisticated, nonlinear transformations of the input variables. If not specified otherwise, the results are reported for classifiers trained only on a single instance in time, *t*, which were the values of the continuous signal resulting from the convolution of the spike trains by the exponential kernel (Figure 1C). The total number of values used to train each classifier depended on the number of trials (see Table S2).

Thus, for most analyses, different classifiers were trained for each time point along the trial, each classifier having its own unique set of weights. These classifiers are denoted as *R_t_* and should be distinguished from *R_int_* classifiers, which were trained to generalize by using only one set of weights to classify neuronal responses over longer periods of time (e.g., 100-ms or 300-ms long). In general, *R_t_* classifiers have better performance than *R_int_* classifiers because a single *R_int_* classifier uses only one set of weights to accomplish a number of different classification tasks, each taking place at a different time point. A corresponding collection of *R_t_* classifiers approaches this problem by sharing the workload—each classifier having its own set of weights optimized for the unique properties of the responses at that time point, *t*. Consequently, the recent history affecting the activity at time *t* imposes fewer constraints on the *R_t_* than on the *R_int_* classifier, the latter having to accommodate variability of response properties over an entire time interval. All classifications were made as binary choices among two stimuli (such as A and D), the chance level for correct classification being 50%. In all graphs, the classification performance of *R_t_* (*R_int_*) readouts is shown as percentage correct classifications of the presented stimulus (see Materials and Methods for more details on experimental and analysis procedures).

### High-Classification Performance and Fading Memory

#### High-classification performance

**Figure 2:**
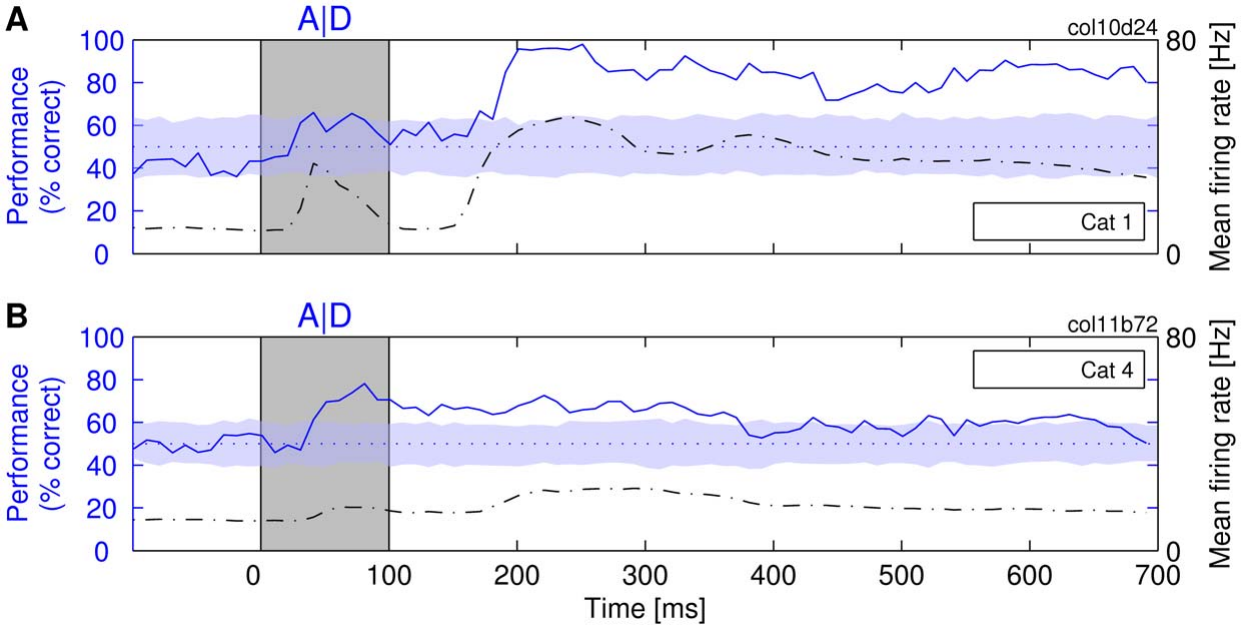
The ability of a linear classifier to determine the identity of the presented letters, A or D. The classification performance is shown (solid line) as a function of time passed from the presentation of the stimulus (at 0 ms) until the moment at which a sample of neuronal activity was taken for training/testing the *R_t_* classifier (the stimulus was removed at 100 ms). Dash-dotted line: the mean firing rate across the entire population of investigated neurons. Dotted line: expected performance at chance level (50% correct). The shaded horizontal stripe around the dotted line covers the region of statistically nonsignificant deviations from the chance level (*p*>0.05), estimated by a label-shuffling test. In this and all other figures, the color of the stripe matches the color of the performance curve for which the statistical test was performed. (A and B) Two different experiments on different cats.

Figure 2 shows an example for the classification performance when a single letter is presented at a time. The classifiers achieved near perfect performance in some experiments (in Figure 2A, close to 100% correct at *t* = ∼200–250 ms), in others, the performance stayed reliably above chance level (Figure 2B). This high-classification performance was achieved despite the high trial-to-trial variability of the responses (see Materials and Methods). The main factor influencing performance was the total number of spikes available for the analysis. Classification was almost always better for datasets with larger numbers of spikes. The classification shown in Figure 2A was based on an average of 1,729 spikes per second (62 units recorded simultaneously), whereas the classification in Figure 2B had to be performed with only 874 spikes per second (49 units) (see Text S1 for more details on predictors of overall classification performance).

This dependence of classification performance on spike counts also held for stimulus-specific changes in firing rate. High rate responses were associated with better classification performance than weak responses. For the cases shown in Figure 2, best classification performance was achieved at the times at which the mean firing rates (Mfrs) had also the highest values. This relationship is most clearly seen in Figure 2A, where the Mfr shows a strong biphasic modulation in response to the on- and offset of the stimulus. The initial increase in discharge rate at ∼50 ms is associated with an increase in classification performance, and the second, much larger increase in Mfr that peaks at about 200–250 ms is associated with the highest level of classification performance (approaching 100%). This correlation between classification performance and Mfr is clearly expressed by a positive value of Pearson's coefficient of correlation (*r* = 0.91) between Mfr and classification. Here, one needs to consider that cortical neurons respond with latencies of 30–60 ms to light stimuli [40]. Therefore, the stimulus-induced changes in Mfrs and the corresponding changes of the classification performance are always delayed relative to the stimuli (which are indicated by gray rectangles in the figures).

In Figure 2B, the biphasic changes in rate responses are much less pronounced than in Figure 2A. Consequently, the changes in classification performance are also less pronounced, the correlation between classification and Mfr remaining positive (*r* = 0.63). The overall correlation for all investigated responses was 0.65. Similar results were obtained for the *R_int_* classifiers (see Figure S1).

#### Long-lasting persistence of information

A remarkable finding was that information about stimulus identity did not disappear quickly after the removal of the stimulus but was often available for an extended period of time. In some cases, the performance stayed above chance level for the entire duration of our trials for which we initially recorded neuronal responses (i.e., 700 ms after stimulus onset for the experiment shown in Figure 2A). The results from the experiment summarized in Figure 2B are similar. Although the classification performance dropped considerably at about 400 ms in this experiment, the performance nevertheless stayed above chance level for a large part of the remaining duration of the trial. Therefore, the information about the nature of stimuli appears to be available as long as the neuronal firing rates stay elevated. Similar long-lasting off-responses in area 17 and under anesthesia have been reported previously ([41], pp. 108–109, 116). These results indicate clearly that the information about stimuli persists long beyond the disappearance of the stimuli, which allows us to investigate the interactions with new stimuli presented after the offset of the first stimulus.

To investigate whether these long-lasting responses were entrained (learned) during the stimulation procedure, we compared the peristimulus time histograms (PSTHs) to the sequences of flashed letters early in the stimulation protocol (e.g., first 10 or 50 trials) with those occurring late (e.g., last 10 or 50 trials). The Mfrs were similar across all subsets of trials and always at least two times larger than spontaneous activity, even 700 ms after stimulus onset (in the case of cat 4, only 300 ms after stimulus onset), indicating that there was no entrainment of these long-lasting responses. Moreover, the duration of the elevated Mfrs in the off-responses were not specific to flashed stimuli, as we observed a similar duration also for off-responses to classical sinusoidal grating stimuli (Figure S2).

### Sequences of Stimuli

#### Responses to a second letter in a sequence

In most of our experiments, we presented not one but a series of three letters. This made it possible to investigate whether new stimuli erase the information about the preceding ones—akin to the masking effect in iconic memory [26],[27],[29],[42]—or alternatively, whether the information about the new stimuli can coexist with and is superimposed on the information about the preceding stimuli.

**Figure 3:**
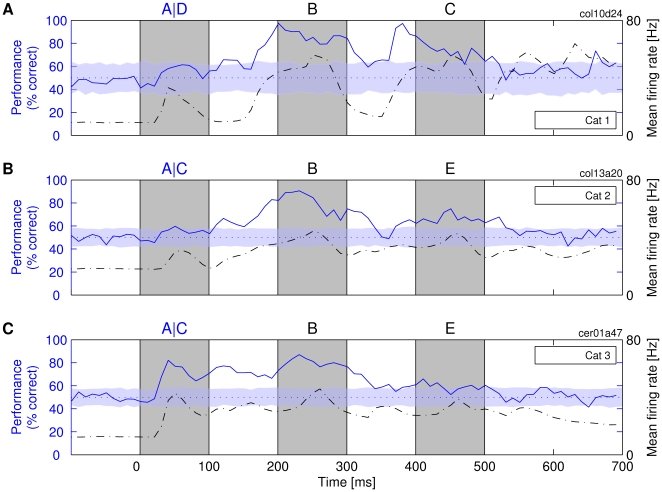
Availability of information about a stimulus presented as a part of a sequence. Classifiers *R_t_* were trained to identify the first letter in the sequences, i.e., ABC versus DBC in one experiment (cat 1) and ABE versus CBE in the other two experiments (cats 2 and 3). (A–C) Three different experiments on different cats. Notations are the same as described in Figure 2.

**Figure 4:**
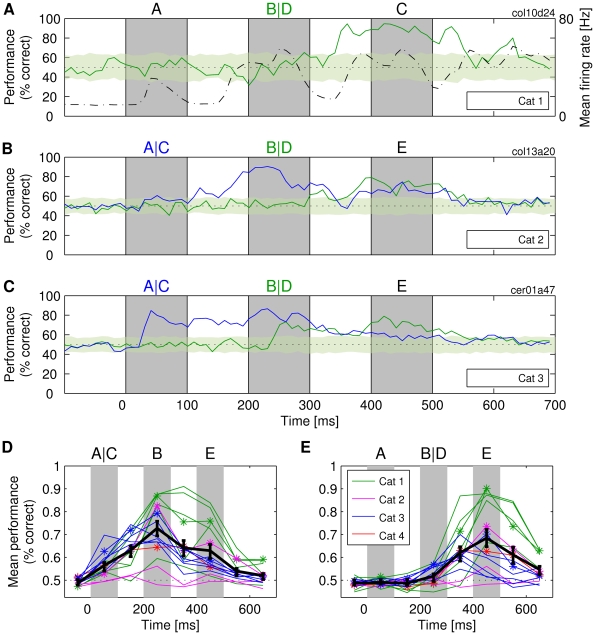
Simultaneous availability of information about multiple stimuli in a sequence. (A) Performance of time-specialized classifiers *R_t_* trained on individual time points to identify the second letter in the sequences of three letters (i.e., ABC vs. ADC). The results should be compared to Figure 3A. (B and C) Simultaneous availability of information about two different letters of a sequence. The following four sequences were presented: ABE, CBE, ADE, and CDE, and two classifiers identified each the presentation of either the first (blue line) or the second letter (green line). Shaded stripe and dotted lines are as described in Figure 2. (D and E) *R_t_* classification performance for 16 different recordings made across four cats. Due to the sparseness of responses, the results are given as average classification performance within 100-ms windows. Thick black curve: gross average across all datasets. Vertical lines: standard error of the mean across all datasets of all cats. Asterisk: the datasets chosen for further analysis.

In all experiments, the classifiers could retrieve the information about the first stimulus in the sequence, not only in the on- or off-responses to that stimulus (i.e., up to 200 ms after its onset), but also in the responses to a later stimulus in the sequence (i.e., from ∼250 ms onward). In some cases, even classification based on the off-responses to the second stimulus approached 100% accuracy for the identification of the first stimulus (Figure 3A, at ∼380 ms). See Figure 4D for classification performance over all experiments made. Thus, when a novel letter was presented, the information about the previously presented letter was not erased but was still present in the responses to the novel stimulus and could be extracted reliably by a linear I&F classifier. This suggests that the second letter in a sequence produces none or only weak masking effects on the information about the first letter.

As with the presentation of single letters, there was again a strong positive correlation between classification performance and Mfr. For the case exemplified in Figure 3A–3C, *r*-values were 0.45, 0.68, and 0.66, respectively (explained variance: 20%–46%; analysis window: 800 ms). Thus, the best classification performance coincided with maximal Mfr. Importantly, however, in the present analysis, the elevated Mfr was evoked, not by the stimulus that was classified, but by another stimulus that was presented later. This suggests that elevated firing rates (i.e., the total spike count) are more important for the readout than the identity of the stimulus that has caused the elevated rates or than the time elapsed since the presentation of the target stimulus. In a control experiment, we could show that the first letter could be classified well even if, as the second stimulus, we presented a blank white screen instead of a letter (Figure S3). Thus, it did not matter whether the Mfr was increased by a structured or by an unstructured stimulus. The role of various factors for coding information in these elevated Mfrs, such as rate responses over longer periods of time (e.g., 100 ms) and temporal structure of action potentials on a shorter time scale (e.g., ≤∼20 ms), is investigated explicitly in “Analyses of Information-Carrying Variables.”

#### Suppressive role of the second stimulus

A comparison between responses to one- and three-letter stimuli (i.e., Figures 2 and 3; for a direct comparison, see Figure S4) suggested that the second stimulus had strong suppressive effects on the off-responses to the first stimulus. This was confirmed by a control experiment in which we manipulated systematically the interval between the first and second stimulus (see Figure S3), where the second stimulus interacted with the dynamics of the off-responses to the first stimulus: The on-response to the second stimulus did not simply sum up with the off-response to the first stimulus. Instead, when presented in temporal proximity (e.g., interspike interval [ISI] = 100–300 ms), the second stimulus clearly produced a suppressive effect on the otherwise strong and sustained off-responses to the first stimulus (i.e., with ISI = 500 ms). Likewise, the properties of the preceding stimulus affected the on-responses to the second stimulus, an effect that was obviously sufficiently specific to classify accurately the first stimulus. Interestingly, we found only very limited evidence that this one-back memory mechanism occurs due to the adaptation of neuronal responses (see Figure S5). Also, the control experiment in Figure S3 was designed to avoid repetitive presentation of stimuli with a single ISI, unlike the other experiments. Thus, this experiment provides additional evidence that the long-lasting off-responses cannot be explained by an entrainment (learning) process that would develop expectancies for a specific ISI (i.e., the rhythm of stimulation).

#### Responses to a third letter in a sequence

The responses to the third stimulus in the sequence (C or E in Figure 3; from ∼450 ms onward) contained much less information about the first stimulus than the responses to the second stimulus. This did not appear to be due to a simple decay of information over time. In one experiment, we were able to compare directly the responses of the same neurons to a single stimulus and to triplets of stimuli (cat 1 in Figures 2A and 3A; direct comparison in Figure S4). With a single stimulus, classification performance was still high at delays as long as 450 ms, but had dropped to chance level at the same time point when sequences of three stimuli were presented. Similar results were obtained in other experiments (Figure 3B and 3C). In contrast to the presentation of one or two letters, information about the first letter was always considerably reduced when a third letter was presented. Also, in the experiment with varying interstimulus interval in Figure S3, the on-responses to the distant second stimulus (300 ms) contained much more information about the first stimulus than any other on-responses to equally distant third stimuli in Figure 3. Furthermore, the reduction in classification performance in third stimulus responses was not due to a reduction of Mfrs. This indicates that the third stimulus in the sequence actually acted as a mask and erased the information about the identity of the first stimulus. Thus, the system has nearly unimpaired memory for one-back but not for two-back stimuli.

Another remarkable finding was that off-responses often returned temporarily to the level of spontaneous activity before again assuming high levels of Mfr (e.g., 100–150 ms and ∼350 ms in Figure 3A; see also Figure S3). This rebound was strongest if the screen was left blank, and hence, no suppression was induced by the presentation of subsequent stimuli. Therefore, the changes in the system responsible for the memory effect have persisted across intervals during which the activity of the neurons used for classification was low and carried little or no information about the stimulus.

### Superposition of Information about Different Stimuli

Responses that carry the information about past stimuli should also carry information about the most recent stimuli, i.e., about those that evoked the responses. The results of the following analyses indicate that this is the case. In Figure 4A, a readout is fed with inputs from the same set of neurons as in Figure 3A (and in Figure 2A), but this time, the readout is trained to classify the second stimulus in the sequence of three letters (letter B vs. D). The classification performance was high (∼80%) despite the high degree of similarity of the shapes. Consistent with our previous findings, classification performance correlated positively with Mfr (*r* = 0.39 for the entire period of 800 ms).

In Figure 4B and 4C, we show results for cats 2 and 3 in which both the first and second letter in the sequence were varied at the same time, allowing us to estimate whether information was available to classify simultaneously both the first and the second stimulus in the sequence. We found that such sequence-specific information was available in the on- and off-responses to the second stimulus. During this period, the classification performance for the first stimulus was not lower than the performance for the recent stimulus that evoked the response (e.g., around 70% correct in both cases for cat 3). We could also show that readouts could be trained to perform nonlinear exclusive OR (XOR) classification, i.e., fire if either a sequence AB_ or CD_ was presented, but not if sequences AD_ or CB_ were presented (for details, see “Nonlinear superposition of information” in Text S1).

In Figure 4D and 4E, we show that these findings generalized across all the cats and, as a rule, also across different recordings made from the same cat. For the classification of the first letter, the gross average maximum performance across the total of all 16 recordings peaked at ∼80% correct during the presentation of the second letter (Figure 4D), and similarly, the classification of the second letter peaked at ∼75% correct during the presentation of the third letter (Figure 4E). In Figure 4D and 4E, one can also see one more remarkable result: classification performance is typically better for off- than for on-responses (e.g., in Figure 4D, the performance is overall higher at 250 ms than at 150 ms). This result can also be seen in Figures 2A, 3A, and 3B (i.e., for cats 1 and 2). The results indicate that successful stimulus classification is a ubiquitous phenomenon and that off-responses contain substantial information about the stimuli even when interacting with on-responses to stimuli presented next in the sequence.

### Analyses of Information-Carrying Variables

The information extracted by the classifier could be encoded either by slow changes in firing rates (slow-rate code; e.g., ∼100 ms) or by precise timing of neuronal spiking events (i.e., fine temporal code; e.g., ≤∼20 ms) or both. Our next step was to investigate the contribution of information encoded at these two different time scales.

#### Information carried by spike timing

**Figure 5:**
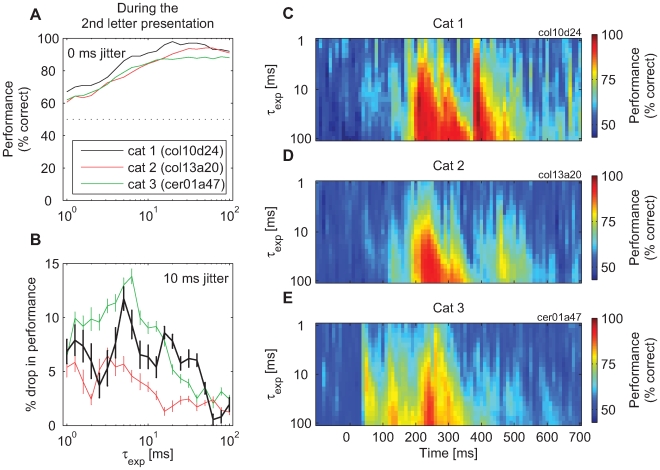
The change in classification performance for *R_t_* classifiers as a function of the time constant, τ. (A) *τ* is changed systematically between 1 and 100 ms (both for training and testing). The performance reached its plateau at about 20 ms. (B) Detrimental effect of 10-ms jitter on the classification performance achieved with different values of *τ*. As a rule, jitter caused a stronger drop in performance with small than with large values of *τ*. However, this function was not monotonic, as the performance drop was strongest when *τ* had values of 5–10 ms. Vertical bars: standard error of the mean across jittered datasets. (C–E) A detailed analysis of the relation between the value chosen for the integration constant, τ (1…100 ms) and the classification performance of *R_t_* readouts. Changes in performance are shown for three cats. As a rule, classification performance increases with longer integration constants.

An analysis of classification performance with τ varying between 1 and 100 ms indicated that, at the points of peak performance, the classification with τ = 20 ms was about as good as with any other larger value of τ, although longer integration constants always led to equivalent or better performances (Figure 5A). At other time points, larger values of τ were required for maximum performance, but these values increased monotonically with the distance from the points of peak performance (the right-skew–shaped intensity plots in Figure 5C–5E). This suggested that an important role of long τ's was to reach sufficiently far into the past in order to “carry over” the responses from the time when they were most informative about the stimulus.

**Figure 6:**
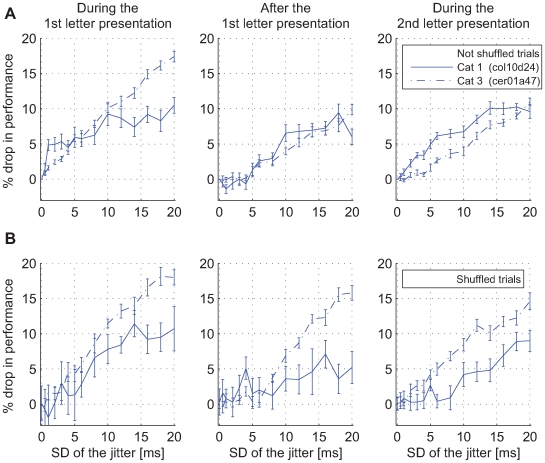
The performance of the classifiers *R_t_* (from Figure 3) as a function of Gaussian jitter applied to spike timing. (A) Change in classification performance as a function of the amount of jitter shown for the three points with the highest classification prior to the application of jitter. The results are shown for three different points in time exhibiting the strongest effects (cat 1: 60,120, and 200 ms; cat 3: 40, 120, and 230 ms). (B) The same analysis as in (A) but for spike trains shuffled across presentation trials (cat 1: 40, 130, and 220 ms; cat 3: 40, 120, and 250 ms). Vertical lines: standard error of the mean across all jittered and trial-shuffled datasets.

We next investigated the role of precise spike timing for achieving such high-classification performance with τ = 20 ms. To this end, we perturbed the fine temporal structure of the spike trains. The times of action potentials were jittered by moving each spike by a random time drawn from a Gaussian distribution with the mean of zero and a prespecified standard deviation (SD) (*x*-axis in all plots in Figure 6; both training and test trials were jittered). We then investigated the ability of readouts to learn to classify the first letter in the sequence based on such jittered datasets (see Materials and Methods for details). Jitter had strong detrimental effects on classification performance as it reduced the performance in all analyses. In Figure 6A, changes in classification performance are shown as a function of the amount of jitter. For this purpose, we selected three time points that exhibited the highest classification performances prior to the application of the jitter and were thus most relevant for such analyses (see Figure S6 for analyses of all other time points and other values of τ). Jitters with standard deviations that were too low to affect slow-rate code still resulted in a considerable performance drop (e.g., jitter with SD = 10 ms typically resulted in a 5%–10% drop in the number of correct stimulus classifications). Often, perturbations of spike timing with SD of only a few milliseconds reduced the performance of the classifiers by a statistically significant amount (e.g., Figure 6A, during the presentation of the first and the second letter).

This detrimental effect of the jitter on the ability of the readouts to classify the stimuli was largely independent of the time in the trial at which the tests were made. The performance was reduced irrespectively of whether the classification was based on on- or on off-responses or whether it was made for the most recently presented stimulus or for the stimuli presented earlier (one-back memory). For jitter with a SD = 20 ms, the average peak performance dropped by 10.7% (see Figure 6A and 6B). Thus, the results indicate that the stimuli are classified most accurately on the basis of the original temporal structure of the responses. This suggests that readouts with short time constants rely less on the information contained in slow firing rates and more on the information about firing probabilities defined within short time windows. This, latter form of information coding can be described as fast changes in firing rates or precise spike timing, and is reflected by narrow and high peaks in the PSTHs, examples of which can be seen in Figure S7 (first row).

The readout was always retrained on jittered data. Consequently, this procedure allows an assessment of the contribution of precise spike timing to classification performance. Hence, the observed drop in performance of ∼10%–15% is an estimate of the proportion of information available through precise timing only. As shown in Figure 5B (and Figure S6), this drop in performance also depends strongly on the choice of the time constant, and is the strongest with τ = ∼5–10 ms. In Figure S8, we show that this is also the range in which jitter has the strongest detrimental effects on the differences in the means of the convolved spike trains across stimuli, and consequently, on the signal-to-noise ratio of the convolved spiking signals. Moreover, the smallest detrimental effects were found for cat 2, and this was also the cat with the smallest effect of jitter on the classification performance. In Figures S9, S10, and S15B, we show that increase in jitter SD has a limited impact, as jitter deteriorates the stimulus classification with τ≤∼20 ms only in the original spike trains and not if the information about the precise timing of action potentials has been previously destroyed.

Performance was also analyzed with shuffled trials. Jitter analysis alone did not provide information on whether the fine temporal structure used by the readouts was time locked to the stimulus events (*evoked* temporal structure) or, alternatively, whether this structure resulted from internal interactions that were not time locked to the stimulus (*induced* temporal structure or *noise correlation*). To distinguish between these alternatives, we randomly shuffled the order of training and test trials for each unit and then repeated the jitter analysis. As a result, the spike trains that readouts received simultaneously from the different input units were in fact not recorded simultaneously but in different, randomly selected trails, which made our analysis more similar to that of Hung et al. [23]. This manipulation preserved stimulus-related information because all the trials were recorded in response to the same stimulus, although the responses for each neuron came from a different trial. Such trial-shuffling procedures destroy the precise temporal structure of internally generated activity patterns (induced temporal structure) but preserve the temporal structure of patterns caused by stimulus locking (evoked temporal structure) [43],[44]. Thus, shuffling is expected to cause a drop in performance if information is contained in the induced (internally generated) temporal organization of responses, and if jitter is applied in addition to shuffling, a further drop in performance would indicate that further information is contained in the evoked (stimulus-locked) temporal structure of the responses.

Trial shuffling produced only occasionally a decrease in classification performance, and these changes were neither consistent across different experiments nor for different time-points along the trial. The time periods showing a significant drop in performance were of very short duration and differed between cat 1 and cat 3 (on average 0.8% and 2.0% drop in performance, respectively; all *p*-values>0.06, signed-rank test, *n* = 30) (see Figure S11). Sometimes, shuffling even increased the classification performance, perhaps by balancing the trial-to-trial variability in neuronal rate responses, which occurs in a highly correlated manner across neurons [24]. These results indicate that *R_t_* classifiers relied only to a small degree on the timing information contained in internally generated temporal patterns (induced temporal structure). Similar results were obtained when, instead of the trial shuffling, we randomly exchanged action potentials among the trials recorded in response to the same stimuli (Figures S12 and S13) while maintaining the same Fano factor for the spike counts as observed in the original data. When jitter was added to the shuffled trials, classification performance dropped consistently and by the same amount as when unshuffled trials were jittered (compare Figure 6B to Figure 6A). These results for *R_t_* readouts indicate that stimulus-specific information is contained in the precise timing of individual spikes and that the relevant temporal structure of the spike trains results from stimulus locking and not from internally generated temporal patterning that would be independent of the temporal structure of stimuli.

#### Generalized information contents across longer stretches of signals

In all the analyses presented so far, the weights of a linear classifier were trained—and the classification performance was tested—on convolved neuronal activity extracted from one specific time point, *t*. As a result, classifiers trained at different time points had different sets of “synaptic” weights, each set being optimized for stimulus classification at exactly one point in time. An analysis of the weight distributions revealed that these weights were highly dissimilar for different time points. The values of weights changed quickly along the trial and even flipped their signs as often as every 50–100 ms. Thus, the optimal set of weights for good stimulus identification differs substantially across different time points.

**Figure 7:**
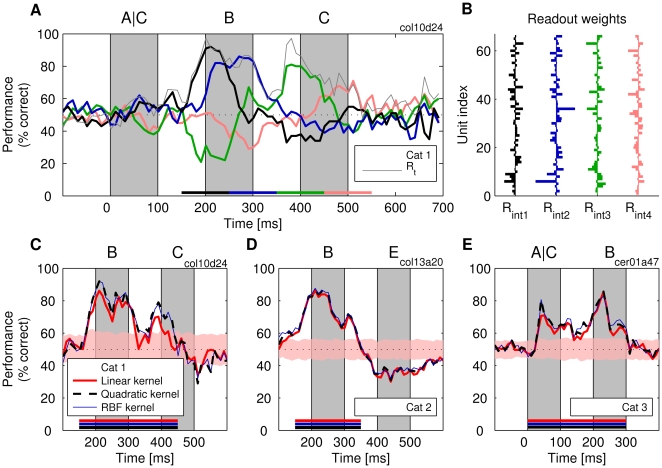
Performance of classifiers *R_int_* trained to extract information during an interval lasting 100–300 ms (τ = 20 ms). (A) Performance for four linear classifiers, each trained for one of the four consecutive intervals of 100-ms duration (color-coded bars). Thin gray line: classification performance of specialized *R_t_* classifiers. (B) The weight vectors learned by the four classifiers in (A). (C–E) The performance of SVM classifiers with different kernels trained on 200-ms (D) and 300-ms (C and E) intervals for three different experiments. Shaded stripe and dotted line are as described in Figure 2.

We also investigated the degree to which our readout systems could achieve good classification performance when trained on longer time intervals. Hence, readouts were trained on all time points within a time interval of either 100-ms or 300-ms duration, and their performance was tested on each time point, *t*. For both training and test, the integration constant remained unchanged, i.e., τ = 20 ms. These readouts were denoted as *R_int_* classifiers or time-invariant classifiers (to distinguish them from time-specialized, *R_t_*, classifiers). In Figure 7A, classification performance is shown for *R_int_* classifiers trained on 100-ms intervals of responses obtained from cat 1, and their weights are shown in Figure 7B. The performance was overall lower than that of *R_t_* classifiers (up to 16% decrease; colored lines in Figure 7A), but nevertheless, at most time points, the classification was above the chance level, the shapes of the performance curves revealing a high degree of similarity to those of the *R_t_* classifiers. Thus, time-invariant classification is also possible. As expected, the performance of the *R_int_* classifiers also dropped to chance level when tested on time points on which they were not trained. Interestingly, readouts sometimes produced even more classification errors than expected by chance. This “rebound” effect is exemplified by the green line in Figure 7A. Here, the classifier was trained on the 350–450-ms interval and tested on time points within the 150–250-ms interval. An analysis of the weights of the *R_int_* classifiers revealed that they were also highly dissimilar when trained at different intervals. For the four *R_int_* classifiers shown in Figure 7A, the average correlation coefficient ranged between *r* = −0.03 (cat 1) and *r* = 0.33 (cat 3). In conclusion, these analyses indicate that training and testing have to be performed on exactly the same time intervals to achieve good performance irrespective of whether *R_int_* or *R_t_* classifiers are used.

Less critical was the duration of the interval for which *R_int_* classifiers were trained. Even with time intervals longer than 100 ms, good classification performance could be achieved. In Figure 7C to 7E (cats 1 to 3), we show results for analyses with 200- or 300-ms-long training intervals. The performance did drop relative to the *R_t_* classifiers (thin gray line in Figure 7A) but stayed nevertheless high above the chance level. As was the case with *R_t_* classifiers, performance of the *R_int_* classifiers was best when Mfrs had high levels. This suggests that the performance of *R_int_* classifiers can increase further and perhaps approach the ceiling of 100% accuracy if the total number of spikes can be increased by recording simultaneously from even more neurons.

In *R_int_* classifiers, the decrease in performance, in comparison to *R_t_* classifiers, was traded off with an increased resistance to jitter. The performance of *R_int_* classifiers was not affected by small jitter (SD = 10 ms). The maximum performance of *R_int_* classifiers dropped by 1% and 3% for cat 1 and cat 3, respectively, and these changes were not significant. Also, the effect of jitter has decreased with the size of the generalization interval, approaching the region of zero effects at interval lengths of about 100 ms (Figure S14). Apparently, as the *R_int_* classifier must generalize over the long period of time, unlike the *R_t_* classifier, it cannot benefit from precise stimulus-locked events that occur at unique time points along the trial. Thus, forcing a classifier with short integration constants to use the same set of weights over the entire duration of the trial prevents these classifiers from relying on precise stimulus-locked information (see Figure S8 for an account of this phenomenon based on signal-to-noise ratios).

The notion that *R_int_* classifiers learned to decode information differently than *R_t_*s is suggested also by the finding that shuffling of trials led to a drop of performance of *R_int_* classifiers. With *int* = 300 ms, the drop in classification performance was highly consistent across different experiments and along the trials, and amounted up to 14% (on average, 4.5% and 6.5% for cats 1 and 3, respectively; all *p*-values<0.0001, signed-rank test, *n* = 30). In no case did shuffling improve performance as observed with *R_t_* classifiers in cats 1 and 3 (Figure S15). As the performance of *R_int_* classifiers did not depend on jitter, this result suggests that these classifiers take advantage of correlations that occur on longer time scales (i.e., covariation in slow-rate responses).

#### Nonlinear readouts and the important role of second-order correlations

Finally, we investigated whether classification performance could be enhanced when the classifiers performed nonlinear operations on the input signals. To this end, we applied SVMs with quadratic kernels or with kernels such as radial basis functions (RBF) that provide high flexibility in implementing nonlinear transformations. Classification performance of these nonlinear classifiers was investigated separately for *R_t_* and *R_int_* readouts and compared to that of linear readouts.

We found no evidence that nonlinear classification improved readout performance of *R_t_* classifiers. Performance improvement never exceeded 2% and never reached the level of statistical significance. Thus, consistent with the report by Hung et al. [23], linear classification was as good as nonlinear classification. This was also true for *R_int_* classifiers if these were tested on responses to single, isolated stimuli, as those shown in Figure 2 (Figure S1).

In contrast, nonlinear *R_int_* classifiers (*int* = 150–450 ms) achieved better performance than linear *R_int_* when applied for the identification of one-back stimuli. Already with quadratic transformations, the ability to identify a previously presented stimulus increased the maximum performance by ∼6% in cat 1 and by 10% in cat 3, but not in cat 2, which was an exception to that result (Figure 7C–7E). On average, performance increased by 5.3% and 2% for cats 1 and 3, respectively, and these changes were significant (two sample *t*-tests, *n* = 10, all *p*-values<0.01). Quadratic transformations use information stored in second-order correlations between the variables more efficiently than linear classifiers, as quadratic operations involve multiplicative operations between the weighted contributions of the inputs (as opposed to simple, weighted summation in a linear system). This form of nonlinearity only takes into account pairwise correlations. Classifiers based on RBFs—by contrast—take into account also higher-order correlations. Remarkably, application of RBFs did not improve performance above the level attained with quadratic classifiers (Figure 7C–7E) (for more detailed analysis, see Figure S16). This indicates that most of the stimulus-specific information used by the nonlinear *R_int_* readout could be extracted already from second-order correlations.

**Figure 8:**
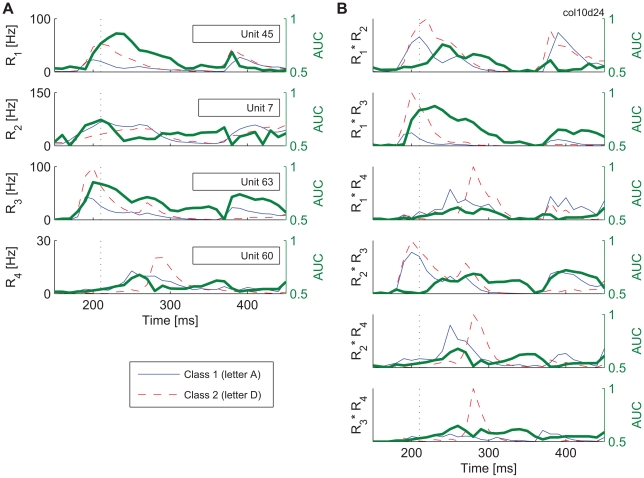
Information contents of neuronal firing rates and their second-order correlations. (A) Left *y*-axis: the average firing rates (Mfrs) in response to two different stimuli for the four most informative units in the analysis of time invariant classification with *int* = 150–450 ms, cat 1. Right *y*-axis: area under receiver-operating characteristic (AUC), related to the probability of correct classification. (B) Pairwise correlations in rate responses, expressed as a product between Mfrs in (A) and the corresponding AUC values obtained from these products.

Previous studies examining synchronization among neuron populations [45],[46] found that second-order correlations can account for almost all the correlation patterns observed in certain natural neuronal networks, and thus, higher-order correlations are unlikely to convey additional information. However, the nonlinear *R_int_* classifiers, being robust to jitter, could not have evaluated precise timing relations between spikes. Instead, they could have relied on slow stimulus-locked changes in firing rates, which may carry information at the level of second-order correlations. To investigate this possibility, for the four input units that made the largest contribution to the performance of the quadratic *R_int_* classifier, we calculated the Mfrs and areas under receiver-operating characteristics (AUC) for all units individually and for the products of the Mfrs for all possible pairs of these units (Figure 8) (see also Figure S17). This revealed that the products differ strongly for different stimuli, leading to high AUC values. Moreover, maximum differentiation between the stimuli occurs often at different time points for products of Mfrs and for individual Mfrs. These results indicate that second-order correlations of neuronal firing rates are informative of stimulus properties and, in principle, can be used by classifiers.

There is one more potential source of correlations that may be picked up by nonlinear *R_int_* classifiers. Averbeck et al. [24] have shown that slow covariations in overall firing rates (responsiveness of neurons) can boost classification performance. In Figure S18, we show that, indeed, firing rates vary along the experiment in a manner that is highly correlated across units. Thus, slow covariations in rate responses may benefit *R_int_* classification when second-order correlations are considered. However, the scope of their contribution is, at best, limited because even if these correlations are removed by shuffling the trials and only stimulus-locked correlations are maintained [43], a polynomial *R_int_* is more efficient than a linear one. Under these conditions, we found a 5.6% and 7.2% increase in the peak performance for cats 1 and 3, respectively. The mean performance along the trial also increased for these two cats by 5.0% and 2.8%, respectively (signed-rank tests, all *p*-values<0.0001, all *n*'s = 30).

In conclusion, these results indicate that time-invariant classification is in principle possible and can attain a very high level of performance if the responses of a sufficient number of neurons can be evaluated. This evaluation can be achieved by linear classifiers similar to I&F neurons. As indicated by the shuffling test, unlike *R_t_* classifiers, the linear *R_int_* classifiers rely to a substantial extent on information that can only be extracted from temporal patterns in the joint activity of the recorded neurons. In addition, nonlinear *R_int_* classifiers, also in contrast to *R_t_*s, can enhance classification performance by using information about correlations, which may come from different sources.

## Discussion

Our results demonstrate that the distributed activity of neurons in cat primary visual cortex (area 17) contains information about previously shown images and that this information is available for a prolonged period of time. The information can be extracted by simple computer-simulated readout neurons and is available for as long as the firing rates stay elevated. These findings are related to the results obtained in macaque IT cortex [23], but there are also a number of differences. First, we show that information required for stimulus classification can be extracted easily from neurons at early processing stages that represent detailed and feature-based information, and under anesthesia. The results are similar to those obtained from neurons in IT that represent categorical information. Second, stimulus-specific information is readily extractable also from responses evoked by the offset of the stimulus (off-responses). This suggests that off-responses play an important role in cortical functions, and hence, should be studied more thoroughly than is usually the case. Third, by presenting sequences of stimuli, we show that the system has reliable memory for one stimulus back. Fourth, we were able to identify the response variables (neuronal code) that carry the stimulus-related information. The classifiers relied on information carried both by neuronal firing rates and by precise timing of neuronal spiking events locked to the stimulus. As the classification was based on an estimate of integration time constants of cortical pyramidal neurons (τ = 20 ms), the results suggest that upstream projection neurons in the cortex—those that receive information from early visual areas—should also be able to use this information. In other words, the results suggest that precise timing of action potentials complements the information available in neuronal firing rates and thus that both types of information can in principle be used as a neuronal code.

The finding that most of the timing information used by *R_t_* readouts was contained in the stimulus-locked timing of action potentials (evoked temporal structure) and that the internally generated timing information (induced temporal structure; noise correlation) supported only *R_int_* classification is probably due to the prominent temporal structure of the rapidly repeating stimuli (5-Hz stimulation rhythm due to 200-ms period between stimulus presentations). Rager and Singer [47] have shown that when a stimulus is flickered with a similar frequency, cortical responses exhibit resonance phenomena to the periodic nature of the stimulus. As a consequence, higher frequencies (>20 Hz) are strongly suppressed. Therefore, for the present stimuli, most of the classification performance based on precise spike timing can be accounted for by inhomogeneous Poisson processes with fast fluctuating rates (≤∼20 ms), whose timing is locked to the stimulus onset. This suggests that neuronal synchrony driven by internally generated rhythms does not play an important role for these stimuli and is in agreement with the finding that if *R_t_* readouts were endowed with polynomial kernels, optimal for detection of pairwise correlations, classification results did not improve. The present results do not allow us to identify the type of information used by real readout systems in the brain. Some cortical neurons may operate with longer time scales (e.g., ∼100 ms) and may rely predominantly on slow changes in rate responses. As our results suggest, these neurons can also achieve good classification performance.

The present results were surprisingly consistent across the range of classifiers used. We obtained similar classification performance irrespective of the type of kernel applied to SVM, for a wide range of the time constants used for convolution of the spike trains, and across different cats. The robustness of these findings is all the more surprising if one considers the high level of trial-to-trial variability, which is a typical property of neuronal rate responses [40],[48]–[53], and was also found in the present data (see Materials and Methods). Future experiments should investigate the level of robustness achievable for more difficult classification decisions than the binary ones made in the present study.

Because the responses fed to the classifier had been recorded simultaneously, we were also able to examine whether additional information was encoded in non–stimulus-locked correlations between the responses of different neurons. Applying nonlinear readouts revealed that this was indeed the case for *R_int_* classification that takes advantage only of slow-rate covariation. Performance improved over the linear readouts, but importantly, this improvement was limited to pairwise correlations (quadratic kernels). One possibility is that similar to precise neuronal synchrony, no additional information is contained in correlations of higher order also for slow covariation in rate responses. This would be consistent with the report that pairwise synchronizations account for most or all of the entropy within the system [45],[46]. Therefore, the claim that higher-order correlations contain no additional information appears to be generalizable to slower time scales.

As *R_t_* readouts operate, by design, with less input information than *R_int_*s, we cannot rule out the possibility that the effects of the second-order correlations (but also those of trial shuffling) would not become significant with a larger dataset—i.e., if more statistical power was available. However, irrespective of this significance, the absolute magnitudes of these effects were always smaller for *R_t_* than for *R_int_* classifiers, whereas on the other hand, *R_t_*s were overall more accurate than *R_int_*s. Thus, a principal difference in the nature of information exploited by the two classification strategies remains and needs to be dealt with. In the present study, we deciphered only partly the characteristics of the two approaches to decoding information. Further efforts, possibly relying heavily on theoretical analyses, will also be needed in order to understand fully the respective implications and (dis-)advantages of the two strategies. Thus, not only the time span of integration (as investigated here), but also the time span (possibly longer) appropriate for generalization, may need to be considered in future studies. These considerations may be relevant for settling the long-standing disputes over the principles by which information is encoded and extracted in the brain (e.g., [31],[54]).

Early visual areas are likely candidates for the implementation of visual persistence and iconic memory [27], because of their retinotopic organization and their noncategorical, feature-based responses [1]. Iconic memory is believed to operate on the basis of automatic (preattentive) processes and it is possible, therefore, that the respective mechanisms also remain operational under anesthesia. Considering that iconic memory declines with similar time constants as the retrievability of stimulus-specific information in the present experiment and is sensitive to masks [26],[27],[29],[42], it is conceivable that we studied a related mechanism. The discovered one-back memory may be responsible for both the integration process in iconic memory and masking effects. The particular outcome would then depend on whether the two subsequent component images (i.e., the “icons”) are meaningful only when isolated (masking) or only when integrated.

Our results suggest that the mechanisms responsible for the temporary storage of stimulus-specific information involve modifications that are often not directly detectable in population rate responses due to the suppression induced by subsequently presented stimuli. Nevertheless, through this suppression processes, the activity from the past affects also the responses to the most recent stimuli. As a consequence, it is possible to identify accurately the past stimuli from the responses evoked by the present. The suppression of sustained activity is likely to involve active inhibition but possibly also more complex mechanisms. Information about the nature of the first stimulus could be contained in the patterning of inhibitory influences, which could then shape the subsequent excitatory responses, but the process may also involve reentry loops from higher cortical areas. Other possibilities are mechanisms of short-term plasticity such as use-dependent transient changes of transmitter release [55], receptor sensitivity [56], and postsynaptic excitability [57]. Although we did not find evidence that adaptation of neuronal responses [58] is responsible for one-back memory, we cannot completely rule out the possibility that these mechanisms play at least some role. It is currently unclear whether and under which circumstances similar long-lasting responses also occur in the awake state and in other brain areas. In some studies, stimuli presented in IT cortex produced short off-responses [23],[59], but in others, the responses are as long as in the present study [60]. One possibility is that the duration of responses depends on the deployment of selective attention, which is necessarily involved in the working memory tasks applied in such studies [61]. Attention may produce suppressive effects similar to those achieved by a second stimulus under anesthesia. Studies on iconic memory suggest this possibility as focusing attention on one subset of items impedes retrieval of the remaining items from that storage [25].

An important factor for temporal integration of information based on fading memory is that the information about a preceding stimulus is not simply superimposed linearly on that of the most recent stimulus. Rather, these effects should be nonlinear, as revealed by the successful XOR classification. Such superposition of information is also a prediction by computer simulations of generic cortical microcircuits [31]. These results indicate that cortical networks are not confined to operating as a serial processing pipeline, where a sequence of precisely structured processing steps is applied sequentially to each input frame but can, in principle, involve kernel-like processors that fuse information from different time slices in such a way that a simple linear classifier (i.e., the readout) can extract information about the stimulus. The important advantage of processes based on fading memory is that novel complex computations can be implemented if only the readout learns by adjusting the weights of its synapses, as emulated in the present study, whereas the remaining part of the system can stay unchanged. The main prerequisite for the implementation of such functions is that nonlinear interactions are combined with fading memory for recent inputs. As suggested by the present study, these are exactly the properties of responses in early visual areas. Thus, cortical neurons at later processing stages have access to signals that have already been subjected to temporal integration.

In conclusion, our data provide support for the existence of fading memory and nonlinear integration between past and present input already at the level of V1. This suggests that the brain exploits the computational advantages of such processes as predicted by computer simulations of generic cortical microcircuits [31],[62], not only for high-level computations, but with all likelihood at all levels of cortical processing. Another, potentially important implication of this finding is that relatively simple readout devices can be used to evaluate complex trajectories of time-varying neuronal activity patterns. This feature can be exploited for the construction of prosthetic devices controlled by neuronal activity.

The present study leaves several questions unanswered. To determine the boundary conditions for the present phenomena, stimulus presentation times need also to be changed systematically. Experiments with larger numbers of electrodes are required in order to examine whether the present findings still hold when the number of simultaneously recorded neurons approaches more closely the number of presynaptic inputs to a typical cortical neuron. Further, psychophysical experiments will be needed to validate our proposal that the present memory effects are closely related to iconic memory. Finally, one would like to obtain direct evidence that cortical neurons actually use the information in the same way as our artificial readouts. However, at present, it is unclear how this could be achieved.

### Concluding Remarks

Olshausen and Field [63] have argued recently that fundamental aspects of visual cortex functions are still unknown. The present results support this notion in that they force us to extend classical theories on visual processing. These are based on the notion that the main functions of the visual processing hierarchy consist of the extraction and recombination of elementary visual features in divergent–convergent and hierarchically organized feedforward architectures (e.g., [37]). In this case, scene analysis is essentially based on frame-by-frame computations in which each new input is processed independently of the previous one. Hence, there is no memory for the past, and such memory is even considered disturbing rather than useful. In contrast, we find evidence for fading memory in combination with nonlinear temporal interactions, at least in early visual areas. Processes supporting both perception and action may benefit from such memory mechanisms because these offer a much wider spectrum of (time-dependent) computations than feedforward architectures. As our data suggest, precise timing of action potentials plays an important role in these processes, suggesting that, besides firing rates, cortical processing also exploits time and temporal relations as coding space.

## Materials and Methods

### Experimental Procedures

All the experiments were conducted according to the guidelines of the Society for Neuroscience and the German law for the protection of animals, approved by the local government's ethical committee, and overseen by a veterinarian. In five cats, anesthesia was induced with ketamine and maintained with a mixture of 70% N_2_O and 30% O_2_ and with halothane (0.4%–0.6%). The cats were paralyzed with pancuronium bromide applied intravenously (Pancuronium, Organon, 0.15 mg kg^−1^ h^−1^). Multiunit activity (MUA) was recorded from area 17 with multiple silicon-based 16-channel probes (organized in a 4×4 spatial matrix), which were supplied by the Center for Neural Communication Technology at the University of Michigan (Michigan probes). The intercontact distances were 200 µm (0.3–0.5 MΩ impedance at 1,000 Hz). Signals were amplified 1,000× and, to extract unit activity, were filtered between 500 Hz and 3.5 kHz. Digital sampling was made with 32 kHz frequency, and the waveforms of threshold-detected action potentials were stored for an off-line spike sorting procedure. The probes were inserted approximately perpendicular to the surface of the cortex, allowing us to record simultaneously from neurons at different cortical layers and at different columns. Up to three probes were inserted simultaneously, allowing for recordings from up to 48 electrode contacts (channels) in parallel, each providing a signal with multiunit activity. This arrangement produced a cluster of overlapping receptive fields (RFs), all RFs being covered by the stimuli (Figure 1A in the main text). Results for one cat were obtained from the same preparation as in [9] and for another cat from [4]. These studies also describe further details on recording techniques.

Stimuli were presented binocularly on a CRT monitor (21″, HITACHI CM813ET) with 100-Hz refresh rate and by using the software for visual stimulation ActiveSTIM (http://www.ActiveSTIM.com). After mapping the borders of the respective RFs, binocular fusion of the two eyes was achieved by aligning the optical axes with an adjustable prism placed in front of one eye. The stimuli consisted of single white letters that spanned approximately 5° of visual angle and that were composed of elementary features suitable for evoking strong responses in area 17. The stimuli were presented with maximal brightness on a black background (thus, also with the maximal contrast) and were placed such that they stimulated the cluster of RFs near optimally. For presentation of single letters, we used letters A and D, each presented for 100 ms. Stimulus sequences of three letters consisted of letters A, B, C, D, and E and were either presented as sequences ABC, DBC, and ADC (cat 1) or sequences ABE, CBE, ADE, and CDE (cats 2 and 3). Each member of a sequence was presented for 100 ms, and the blank period separating the presentation of letters also lasted, in most cases, 100 ms. For each stimulation condition (single letter or a sequence), 50 (cat 1), 100 (cat 4), or 150 (cats 2, 3, and 5) repetitions were made. In a control experiment, we varied the blank period between two stimuli presented in a sequence, the periods having the values 100, 200, 300, and 500 ms. In this experiment, the first stimulus in the sequence was always a letter (A or C), whereas as the second stimulus, we randomly exchanged a letter (B) with a white blank screen that had the same luminance as the letter stimuli. This control experiment was made on a separate cat, which ensured that we fully avoided repetitive presentations of identical blank periods, which were made in other cats. The block randomization of the presentation order ensured that no more than two blank periods of identical duration occurred in a row. Sinusoidal gratings were presented with full contrast, spanned 18° of visual angle, had spatial frequency of 2.6 cycles/degree, and drifted with the speed of 2.0°/s, thereby being suitable for evoking strong responses in area 17. For an overview of experiments performed on each cat, see Table S1.

### Data Analysis

#### Off-line spike-sorting procedures

To extract single units, extracellularly recorded spike waveforms of each channel were subjected first to principal component analysis and then to clustering procedures whereby the waveforms of similar shapes were assumed to be generated by the same neuron [64]. In some cases, separation of single units was not possible due to undistinguishable shapes of waveforms, usually of small amplitude. In this case, multiple single units were pooled into a larger multiunit. Also, it was not possible to treat clearly distinguishable single units as separate entries if these units had too low firing rates. Units with low firing rates have very sparse high-dimensional data representations that can produce weak generalization capabilities of trained readouts (the overfitting problem). Thus, we pooled low-rate units (∼<10 Hz) into a larger multiunit. As a result, we combined on average 2.30 units per recording channel (range one to five units), with about 50% of them being multiunits. In the five recording sessions from the five cats we used, 66, 78, 52, 124, and for one analysis, 25 units of simultaneously recorded activity for training and testing classification (for cat 5, only 13 electrode channels were responding). There was no selection of units for further analyses. Further pooling of units always reduced the peak classification performance. In three cats for which we investigated the effect of pooling beyond the removal of low-rate units (cats 1, 2, and 3), on average, the additional pooling reduced the peak classification performance by 8.8% as compared to the datasets actually used for the analyses (standard deviation across all comparisons was 6.1%). Only for cat 2, this decrease in performance was not statistically significant, and its magnitude was smaller than 1%.

#### Variability of responses

All responses showed a considerable level of trial-to-trial variability. The Fano-factors were computed according to Fano [65] for the total spike counts obtained per recording electrode and across individual stimulation trials within the time interval of 1.2 s, beginning 500 ms before the stimulus onset. The obtained values were on average about 8 and ranged between 1 and 60, and these values were consistent with previous reports for comparable window sizes [66].

#### Classification

The decay time constant *τ* (20 ms in most analyses) was used for convolving the spike times, which means that without further inputs, the “depolarization” of the artificial integrate-and-fire (I&F) neurons (which did not implement a reset or a refractory period) drops within this period with the time course exp(−*t*/τ) to 37% of its initial value (hence the name “leaky I&F”). This convolution endowed the readout neurons with memory for the history of activation (inputs), the duration of the memory being determined by the constant, *τ*. Thus, the resulting activation at any time, *t*, was a function of the current activation and the activation from the recent past, as determined by the speed of the depolarization leak. There were no other mechanisms for the interaction with past inputs. Hence, the dimensionality of the inputs to SVMs learning *R_t_* classification equaled always the number of units (neurons) that participated in the analysis. Note that our implementation of readouts is different from that used by Hung et al. [23], who simply counted the number of spikes that were fired by each neuron within a time bin of 50 ms. We opted for leaky integration because this approach, although resulting in low-pass filtering, nevertheless preserves much information about precise temporal relations between action potentials (time delays). This approach also approximates more accurately the conditions under which real cortical (readout) systems should operate. Nevertheless, we also made analyses with spike counts similar to those reported by Hung et al., and the results remained similar (Figure S19). The spike count yielded roughly the same information as low-pass filtering when the time constant of the filtering corresponded to the time bin used for the spike count. The time constant *τ* can be thought of representing the temporal properties of the membrane and of the synaptic receptors. No additional time constants were used to mimic decays in synaptic currents, and hence, these inputs were modeled as Dirac delta functions. Although classification was based on the linear combinations constructed from input intensities and “synaptic” weights, the final classification also involved one nonlinear step due to the threshold for generation of a “spike” (with exception of the analysis of XOR classification function). The threshold was necessary to make the binary decisions for the presented letters (e.g., letter A or B). The value of this threshold was optimized for maximum performance and determined by the same training procedures as those used to optimize the synaptic weights. All the calculations for spike-timing convolutions were made in steps of 10 ms. In all analyses, the testing was made with the same value for *τ* as the training. Note that the present analyses do not investigate information that may be stored in ISIs.

#### Support vector machine

A support vector machine (SVM) is a supervised learning method for binary classification problems. The training samples are first mapped into a N-dimensional feature space, and classification is performed in this feature space by constructing a hyperplane that optimally separates the data into two classes. The constraint is that the shortest distance from a training sample to the hyperplane (i.e., geometric margin) must be maximized. Consequently, SVMs are not based on iterative procedures. The main advantage of SVMs is that even if the training samples are not linearly separable in the input space, they might become linearly separable in the feature space after an application of a nonlinear feature mapping procedure. Another important property of SVMs is that this nonlinear mapping into the feature space does not need to be defined explicitly provided that the dot product in the feature space can be replaced by a so-called kernel function. In case of kernels based on RBFs, N is even infinite dimensional. We used the LIBSVM library for support vector machines [67]. The classifiers were trained with linear-kernel SVMs, and overfitting was controlled by choosing the parameter C to be 10 in case of 50 repetitions per stimulus condition, and 50 in case of 150 repetitions per condition. The parameter C was optimized prior to the analysis on an independent validation set that was not used later either for training or testing. This parameter sets the tradeoff between minimizing the number of training samples located on the wrong side of the margin on the one hand, and maximizing the geometric margin between correctly classified samples and the classification hyperplane on the other hand [68],[69]. In case of RBF kernels, the variance of the Gaussian kernel was set to 0.5. The classification performance was estimated with 10-fold cross-validation in which we balanced the number of examples for the training and for the test class: For each of the 10 tests, a different set of 1/10 of the data was chosen for testing under the constraint that all 10 test sets were disjoint. For example, for 50 trials, each test was made on another set of five trials, the remaining 45 trials being always used for training. Ten-fold cross-validation was repeated 10 times with different data points in different splits (results represent average values), except for the results reported in Figure 5C–5E and Figure S16, for which, due to the computational time constraints, only a single cross-validation run was carried out. All the reported performance results are averages for the test data. For the number of data points in each analysis, see Table S2. To assess the significance of classification performance, we obtained the H0 distribution for classification performance by randomizing the information about the identity of the stimuli inducing the responses and repeating this randomization procedure 100 times (i.e., a label-shuffling procedure). The sets of four units with best classification were found by determining exhaustively the performance for all possible four-unit combinations. *R_int_* classifiers were trained and classified on all time points of the same 100-ms or 300-ms intervals, classification performance being tested at each 10-ms time point. The significance in the Figure S16 was estimated by performing a two-sample *t*-test of the hypothesis that the two sets of cross-validation errors obtained for the *R_t_* and the *R_int_* classifier come from distributions with equal means (critical *p*-value = 0.05). The significance levels for trial-shuffling experiments was assessed by applying a sign-rank test to the readout decoding accuracies pooled from all 30 time points within the specified time interval. The comparisons were made either between trial-shuffled and not trial-shuffled data or between linear and polynomial readouts trained on trial-shuffled data. To ensure the statistical independence of the differences in performance, at each of the 30 time points, different trial-shuffled datasets were used for training and for testing.

It is important to note that differences in the absolute values of the classification performance (% correct) cannot be used as a direct indicator of the reliability of the reported findings or of the general ability of the readout neurons to extract information. This is because classification performance depends on a number of factors such as the total number of units recorded simultaneously and the total number of action potentials recorded. The performance is necessarily low with fewer electrodes (fewer action potentials), and if the number of units recorded can be increased further (e.g., >100 recording channels), the performance is likely to saturate at 100% correct.

#### Mutual information

We performed also an alternative data analysis based on an information-theoretic approach. Thus, instead of percentage correct classification, we calculated the mutual information between the stimulus properties and the responses of readout neurons. These analyses yielded identical results to those reported for performance correct (for an example of such analysis, see Figure S20).

#### Total firing rates

The average level of activity for the investigated units is shown in all figures as mean firing rate (Mfr). Mfr provides information very similar to that of peristimulus time histograms (PSTHs) (shown only in Figure 1B), but instead of simple spike counts, we show the averaged spike trains after the convolution with the exponentially decaying kernel. Thus, the plots provide the information that is most relevant for the present analyses. The two datasets with lowest classification performance in Figure 4D (purple lines) had weakest responses to visual stimulation, the signal-to-noise ratio of rate responses being ≤1.9. For all other recordings, this ratio ranged between 4 and 12.

#### Jitter analysis

Spike times were always jittered 100 times by adding a value drawn from a Gaussian distribution with a mean of zero and the specified standard deviation (SD). The performance for shuffled and nonshuffled trials was compared for the time points at which the performance was maximal prior to the jitter analysis. Note that these time points were not necessarily identical and were shifted by up to 20 ms (Figure 6). In these analyses, both training and testing were made on jittered spike trains. The results were similar for all three cats, but the performance difference between linear and nonlinear *R_int_* readouts was somewhat smaller for cat 2 (3%–7%) than for other cats.

#### Nonlinear superposition of information

To investigate nonlinear superposition of information in brain responses, we removed the nonlinear component of the classification process, i.e., the activation threshold determining whether classifiers fired or not. For more detail, see Text S1 and Figure S21.

## Supporting Information

Figure S1 **Single letters classification by time-invariant readouts.** Analysis similar to that in Figure 2 of the main text, but instead of using time-specialized classifiers (*R*,), the classification was made on the basis of time-invariant classifiers (*R_int_*) trained on all time points within an interval that had the duration of either 500 ms (cat 1) or 400 ms (cat 4). This analysis was made with three types of SVM classifiers that had either linear or polynomial kernels or kernels based on radial basis functions.
(0.05 MB EPS)

Figure S2 **Long off-responses are not unique to flashed stimuli.** Comparison of mean firing rates (Mfrs) in off-responses observed after removing either the last stimulus in the sequence (blue line) or a sinusoidal grating presented previously for 4 s (red line). The time at which the stimuli were removed is indicated on the abscissa by 0 ms. The duration of the off-responses is similar for the two types of stimuli.
(0.05 MB EPS)

Figure S3 **Variation of the interstimulus interval.** Classification performance in a control experiment in which interstimulus interval was varied (100, 200, 300, and 500 ms), and as the second stimulus, either a letter B was presented (blue solid line) or the screen was blank, i.e., white (w.s. stands for white screen) during this short period of time (green solid line). If good classification performance was evoked only by structured letter stimuli and not by a white screen, the result would have suggested that classification relied on stimulus-specific processing occurring only within this brain area. However, the results indicate that good classification performance was obtained also from rate responses evoked by a white screen. Thus, readout neurons identify efficiently the past stimuli irrespective of the mechanism by which the Mfrs are elevated and are able to take advantage of any type of interaction that occurs between past and present stimuli.
(1.00 MB EPS)

Figure S4 **Direct comparison between one- and three-letter sequences.** The results from Figures 2 and 3 obtained from the same cat are shown here together to facilitate direct comparison. The plot indicates the suppressive effects of the subsequent stimuli in the sequence.
(0.05 MB EPS)

Figure S5 **Adaptation of neuronal responses plays little or no role in the one-back memory for stimulation sequences.** If adaptation plays an important role in the one-back memory phenomenon, it is expected to find negative correlations between the weights of *R_t_* readouts trained to classify the first stimulus early and late in the trials. Also, the rate responses to later stimuli should be reduced if they were high in response to earlier stimuli. (A–C) Cross-correlation matrices of the weight vectors assigned to *R_t_* units for the classification of the first stimulus when all three stimuli are shown. The correlations indicate the vectors' similarities/differences across time. Periods with high-classification performance tend to be correlated positively. The strongest negative correlations were found for the periods in which the classification performance was low (≥∼400 ms) and in the cat that had overall lowest performance (cat 2). Very similar plots were obtained when, instead of vector similarities, we plotted the classification performance at one time based on the vectors trained at another time (unpublished data). The autocorrelations along the diagonal are set to zero (green color). (D and E) A test of adaptation based on a comparison of rate responses within and between letter-sequences. To make comparisons between sequences, quotients of rate responses, *Q*, to two different stimulation sequences (ABC or DBC [D], and ABC or ADC [E]) are computed for each unit and across different time intervals during which either the first (0–100 ms), the second (200–300 ms), or the third (400–500 ms) letter was presented. In (D), *Q* (0–100 ms) = *spike count* (*ABC*)/*spike count* (*DBC*), during the interval 0–100 ms and *Q* (200–300 ms) = *spike count* (*ABC*)/*spike count* (*DBC*), during the interval 200–300 ms. Similarly, in (E), *Q* (200–300 ms) = *spike count* (*ABC*)/*spike count* (*ADC*), for the period 200–300 ms and *Q* (400–500 ms) = *spike count* (*ABC*)/*spike count* (*ADC*), for the period 400–500 ms. Values >1.0 indicate that the responses were higher in the first than in the second sequence. To make comparisons within sequences (at different times), the quotients are presented in a scatter plot. In case of adaptation, one would expect a negative correlation between the values of *Q* early and late in the trial (e.g., in response to the first and second letter). The scatter plot diagrams indicate in most cases lack of such negative correlations between the quotients (the values of Pearson's correlation coefficients, *r*, are not significantly different from zero) with the exception of the classification of the second letter for cat 2 and cat 3 with *r* = −0.55 and *r* = −0.31, respectively. Thus, if a unit is activated more strongly by A than by D early in the trial, this does not predict that the response to letter B, presented later in the same trial, will necessarily be weaker when preceded with A than when preceded with D. The quotients are computed for each unit and are averaged across all presentation trials.
(0.18 MB EPS)

Figure S6 **A detailed analysis of the effect of jitter on the classification performance.** The effect of jitter was calculated along the entire trial and for three different values of the integration constant, τ: 5, 20, and 80 ms. Color code: the amount of jitter (0–20 ms). Jitter always exhibited strongest detrimental effects with τ = 5 ms.
(0.20 MB EPS)

Figure S7 **The effect of jitter on PSTHs.** Precise temporal information was removed from the spike trains by jittering the spike timing with varying standard deviations (SD = 0–50 ms). (A and B) Example PSTHs that gradually lose their fine temporal structure (i.e., become smoother) calculated for one unit in response to two different stimulation sequences (ABC and DBC).
(0.49 MB EPS)

Figure S8 **Analysis of the signal-to-noise ratio in the convolved signals that serve as inputs to classifiers.** (A) Detailed analysis of the two factors that determine the signal-to-noise ratio is shown for cat 1. First row: standard deviation (SD) across all the convolved values that entered the analysis (both training and testing). For *R_t_* classifiers (left column), one SD was computed for each time *t*, which were then averaged across all times *t* that entered the analysis (the same intervals are used as for *R_int_* analysis). In contrast, for *R_int_* classifiers, only one SD value was computed across the entire investigated interval (the same intervals are used as in Figure 7). Second row: absolute mean difference in the rate responses (|Δ *Mean*|) for the two stimuli that needed to be classified. The values |Δ *Mean*| were computed first for each time point *t*, and subsequently averaged across all times points that entered the analysis. (B–D) Signal-to-noise ratio (SNR) defined as SNR = |Δ *Mean*|/SD and shown separately for three different cats. All estimates were made for different integration constants, τ (5–160 ms; *x*-axis) and different amounts of Gaussian jitter (standard deviations: 0–80 ms; color coded). Increase in τ reduces both SD and |Δ *Mean*|, resulting in relatively small changes in SNR. These effects of τ are similar on the input statistics for *R_t_* and *R_int_* classifiers. In contrast, jitter affects strongly only |Δ *Mean*| of the input statistics, but not SD, and only for *R_t_* classifiers. Also, the effects are strongest for the smallest values of τ. Consequently, the effect of jitter on SNR is restricted to *R_t_* classifiers, which are affected most strongly when τ is small. Vertical lines: standard error of the mean across electrodes and time points.
(0.10 MB EPS)

Figure S9 **The classification performance of readouts applied to jittered spike trains (SD = 50 ms) as shown in Figure S7.** (A–C) Classification performance in three different experiments (blue solid line). The performance is lower than that for nonjittered spike trains in Figure 3 of the main text (here shown again by gray solid lines). Nevertheless, the classification performance exceeds the chance level. Dash-dotted line: the mean firing rate across the entire population of investigated neurons before jittering the spike trains.
(0.08 MB EPS)

Figure S10 **No information is encoded in the precise spike timing of previously jittered spike trains.** (A and B) The effect of additional jitter and change in *τ* for the classification performance estimated for the spike trains previously jittered with SD = 50 ms as in Figure S7. (A) Accuracy for classifying the first letter in the sequence as a function of the time constant, *τ*. The performance for *τ*≤∼20 ms is considerably lower than for the trains without the previous jitter, shown in Figure 5. (B) Further application of a small jitter (SD = 10 ms) does not affect the classification performance for any value of *τ*. This is in contrast to previously nonjittered spike trains shown in Figure 5B. (C) Analysis identical to that in Figure 6A in the main text but applied to the spike trains with the same jittered spike trains as in (A and B). Vertical lines: standard error of the mean across jittered datasets.
(0.12 MB EPS)

Figure S11 **Classification performance for** ***R_t_*** **readouts after the application of trial shuffling.** (A and B) Results for cats 1 and 3, respectively.
(0.04 MB EPS)

Figure S12 **Performance of classifiers trained on surrogate data.** Classification performance computed on a set of surrogate data created by using a different procedure for removing information from spike trains than that used in Figure S10. Here, we randomly exchanged the spikes of the same unit across different presentation trials of the same stimulus, while maintaining for each unit the Fano factors for the spike counts. Unlike the jitter, this procedure preserves each spike's latency but removes the associations to the original trials, which in turn removes the dependencies between different spike trains generated by the internal, not stimulus-locked, timing mechanisms (similarly to the trial-shuffling procedure presented in Figure 6B in the main text). After the application of the present procedure, the spike trains correspond to inhomogeneous Poisson processes, which, in the same time, retain the rate profiles of the original PSTHs. (A to C) Classification performance in three different experiments (red solid line). Blue solid line: the classification performance without exchanging the spikes, shown originally in Figure 3 in the main text. Dash-dotted line: the mean firing rate across the entire population of investigated neurons. Vertical lines: standard errors of the mean across surrogate datasets.
(0.19 MB EPS)

Figure S13 **Jitter analysis for surrogate data.** Jitter-based analysis of the spike trains with randomly exchanged spikes across trials as shown in Figure S12. (A) The effect of a small jitter (up to 20 ms) on the classification performance was tested by using a procedure identical to that shown in Figure 6A in the main text (e.g., τ = 20 ms). Small jitter reduced the classification performance in a manner similar to that observed in the original data, supporting the conclusion that most of the fine temporal information used by the readouts was time locked to the stimulus onset (or offset). (B) The effect of small jitter after the fine stimulus-locked temporal structure was first destroyed in the spike trains by applying a large jitter of SD = 50 ms (as in Figures S7, S9, and S10). Vertical lines: standard error of the mean across jittered surrogate datasets.
(0.07 MB EPS)

Figure S14 **Jitter affects only the performance of** ***R_t_*** **classifiers.** Analysis of the drop in classification performance induced by a jitter (SD = 20 ms) shown as a function of the time interval over which the readout was trained. With a time interval = 0 ms, the analysis corresponds to *R_t_* readouts. Otherwise, we refer to *R_int_* readouts. Thin lines: estimates made for 16 time points along the trial that had the highest classification performance. Thick line: the average across all estimates. Vertical lines: standard error of the mean across jittered datasets. (A–C) Estimates made separately for three different cats.
(0.12 MB EPS)

Figure S15 **Performance of polynomial** ***R_int_*** **classifiers trained on trial shuffled data.** Classification performance after trial shuffling as in Figure S11 but for time-invariant polynomial *R_int_* classification with an interval of 300 ms. (A and B) Results for cats 1 and 3, respectively.
(0.04 MB EPS)

Figure S16 **Dependence of the performance of** ***R_int_*** **classifiers on the length of the time interval and performance improvement of polynomial classifiers.** Classification performance for linear *R_int_* readouts (left column) and the improvement in performance when changing the kernel functions from linear to polynomial (right column), shown for different locations of the generalization interval (its endpoint) within the trial (*x*-axis), and as a function of the size of the interval (*y*-axis). Time interval = 0 ms corresponds to the *R_t_* classifier. Only significant differences are plotted in the right column, significance being determined on the basis of cross-validation errors (see Materials and Methods). White stars: parameter values for the analyses shown in Figure 7C–7E in the main text. The performance of *R_int_* classifiers with intervals ≥∼200 ms is consistently facilitated in all cats by the use of polynomial functions.
(0.06 MB EPS)

Figure S17 **Differences in firing rates correlate with the classification performance.** The time course of classifier performance from Figure 3 of the main text shown together with the difference in the mean firing rates between two stimulation conditions (8-fold magnified; dashed lines in red) in addition to the mean firing rates (dash-dotted lines in black).
(0.06 MB EPS)

Figure S18 **Temporal stability of the recordings.** Analysis of the changes in the neuronal firing rates along the experiment for three cats. Left column: time course of the changes in neural responsiveness along the experiments. Each of the ten trial blocks is an average of 260 (cat 1) or 120 (cats 2 and 3) consecutive trials. Each color indicates one electrode. Right column: correlations in the changes of the overall firing rate responses across the electrodes. Positive correlations predominate (red colors). Cat 2 also exhibits a considerable number of negative correlations (blue colors).
(0.07 MB EPS)

Figure S19 **Comparison of classifiers trained on spike counts.** Our main classification method, based on convolved spike trains, is compared to a method based on simple spike counts. At each time point, a time-specialized linear classifier (*R_t_*) was trained, either on convolved spike trains with an exponential decay (time constant, τ = 20 ms), or on the basis of spike counts obtained for intervals of 5, 20, 50, and 100 ms. When similar time constants are used, spike count produces similar classification performance to the exponential convolution.
(0.04 MB EPS)

Figure S20 **Mutual information.** Performance for the same classification task as in Figure 3A of the main text but this time expressed as the mutual information between the stimulus identity and the responses of the readout (blue solid line).
(0.03 MB EPS)

Figure S21 **XOR classification.** Readouts were trained to make a nonlinear XOR-type detection, e.g., fire if either a sequence AB_ or CD_ has been presented but not if sequences AD_ or CB_ have been presented. Here, the performance was assessed by the point-biserial coefficient of correlation between stimulus identity (binary code) and readout “depolarization” (a continuous variable).
(0.04 MB EPS)

Table S1 **Overview of the experiments made on different cats.** Columns: types of experiments/analyses. Rows: cats. Vertical line (|) indicates the stimuli that were altered (and classified) at the give position in the sequence. X indicates a control experiment in which a white blank screen was presented as the second stimulus. s.g., sinusoidal gratings.
(0.03 MB DOC)

Table S2 **The amount of training data available for each analysis.** Indicated are the numbers of data points in the training set (*number of trials*×*number of time points*). For *R_t_* classifiers, *number of time points* = 1. Columns: figure numbers. (A) Figures in the main text. (B) Supplementary figures.
(0.05 MB DOC)

Text S1 **Nonlinear superposition of information and supplementary theorem.**
(0.12 MB DOC)

## Abbreviations

AUC: area under receiver-operating characteristics
I&F: integrate-and-fire
ISI: interspike interval
IT: inferotemporal
Mfr: mean firing rate
PSTH: peristimulus time histogram
RBF: radial basis function
RF: receptive field
SD: standard deviation
SVM: support vector machine
XOR: exclusive OR
