# Supplementary material for: Distributed Fading Memory for Stimulus Properties in the Primary Visual Cortex
Source: PLoS Biol. 2009 Dec 22;7(12):e1000260. doi: 10.1371/journal.pbio.1000260 (PMC2785877; doi:10.1371/journal.pbio.1000260)
Supplement: Table S1 — Overview of the experiments made on different cats. Columns: types of experiments/analyses. Rows: cats. Vertical line (|) indicates the stimuli that were altered (and classified) at the give position in the sequence. X indicates a control experiment in which a white blank screen was presented as the second stimulus. s.g., sinusoidal gratings. (0.03 MB DOC) [file pbio.1000260.s022.doc]

**Table S1:** Overview of the experiments made on different cats. Columns: types of experiments/analyses. Rows: cats. Vertical line (|) indicates the stimuli that were altered (and classified) at the give position in the sequence. s.g.: sinusoidal gratings. X: A control experiment in which a white blank screen was presented as the second stimulus.

|  | A|D | A|D B C | A B|D C | A|C B|D E | s. g. | A|C X |
| --- | --- | --- | --- | --- | --- | --- |
| Cat 1 | ⦁ | ⦁ | ⦁ |  |  |  |
| Cat 2 |  |  |  | ⦁ |  |  |
| Cat 3 |  |  |  | ⦁ |  |  |
| Cat 4 | ⦁ | ⦁ | ⦁ |  |  |  |
| Cat 5 |  |  |  |  | ⦁ | ⦁ |
