# Supplementary material for: Distributed Fading Memory for Stimulus Properties in the Primary Visual Cortex
Source: PLoS Biol. 2009 Dec 22;7(12):e1000260. doi: 10.1371/journal.pbio.1000260 (PMC2785877; doi:10.1371/journal.pbio.1000260)
Supplement: Table S2 — The amount of training data available for each analysis. Indicated are the numbers of data points in the training set (number of trials×number of time points). For Rt classifiers, number of time points = 1. Columns: figure numbers. (A) Figures in the main text. (B) Supplementary figures. (0.05 MB DOC) [file pbio.1000260.s023.doc]

**Table S2:** The amount of training data available for each analysis. Indicated are the numbers of data points in the training set (*number of trials* × *number of time points*). For *R_t_* classifiers *number of time points* = 1. Columns: Figure numbers. **A**, Figures in the main text. **B**, Supplementary figures.

A

|  | 2 | 3, 4 | 5 | 6 | 7A | 7C-E | 8 |
| --- | --- | --- | --- | --- | --- | --- | --- |
| Cat 1 | 100 | 100 | 100 | 100 | 1000 | 3000 | 3000 |
| Cat 2 |  | 300 |  | 300 |  | 6000 |  |
| Cat 3 |  | 300 | 300 | 300 |  | 9000 |  |
| Cat 4 | 200 | 200 |  |  |  |  |  |

B

|  | 1 | 3 | 4 | 5, 6, 9, 10, 12, 17, 19, 20 | 11, 13 | 14 | 15 | 16 | 21 |
| --- | --- | --- | --- | --- | --- | --- | --- | --- | --- |
| Cat 1 | 5000 |  | 100 | 100 | 100 | 100-2000 | 3000 | 3000 |  |
| Cat 2 |  |  |  | 300 |  | 300-6000 |  | 6000 | 600 |
| Cat 3 |  |  |  | 300 | 300 | 300-6000 | 9000 | 9000 | 600 |
| Cat 4 | 8000 |  |  |  |  |  |  |  |  |
| Cat 5 |  | 300 |  |  |  |  |  |  |  |
