# Supplementary material for: Distributed Fading Memory for Stimulus Properties in the Primary Visual Cortex
Source: PLoS Biol. 2009 Dec 22;7(12):e1000260. doi: 10.1371/journal.pbio.1000260 (PMC2785877; doi:10.1371/journal.pbio.1000260)
Supplement: Text S1 — Nonlinear superposition of information and supplementary theorem. (0.12 MB DOC) [file pbio.1000260.s024.doc]

Supporting information for the manuscript “Distributed fading memory for stimulus properties in the primary visual cortex”

Danko Nikolić ^1,2^ ^*^, Stefan Häusler ^3 *^, Wolf Singer ^1,2^ and Wolfgang Maass ^2,3^

*^1^ Department of Neurophysiology, Max-Planck-Institute for Brain Research, D-60528 Frankfurt (Main), Germany*

*^2^ Frankfurt Institute for Advanced Studies (FIAS), Johan Wolfgang Goethe University, D-60528 Frankfurt (Main), Germany*

*^3^ Institute for Theoretical Computer Science, Graz University of Technology, A-8010 Graz, Austria*

(^*^) These authors contributed equally to this work.

***Non-linear superposition of information***

To achieve high flexibility of feed-forward neuronal computations, it is advantageous if neurons perform non-linear transformations of their inputs (Minsky and Papert 1969). Non-linear superposition of information from subsequent frames of online inputs is an essential ingredient for models that emphasize the dynamical system aspect of the recurrently connected visual system as a whole. Furthermore, it was shown in Natschläger and Maass (2005) to be a direct consequence of generic recurrent circuits of neurons. In terms of machine-learning theory, the functions of recurrent circuitry can be seen as analogues of non-linear kernels of a support vector machine (SVM) (Vapnik 1998; Schoelkopf and Smola 2002). We investigated whether responses in visual cortex have similar kernel-like properties by being able to fuse sequentially arriving pieces of information in a non-linear manner. Non-linear interactions can be assumed if a readout is capable of producing an XOR classification function of the stimulation sequence. In this case a classifier should return one (i.e., a spike) if either a sequence ‘AB_’ or ‘CD_’ has been presented, and should return a zero (i.e., no spike) if sequences ‘AD_’ or ‘CB_’ have been presented. For this analysis we removed the non-linear spiking threshold of the simulated I&F neuron. This manipulation was needed to ensure that all the non-linear transformations necessary to compute the XOR function were made by the brain and not by the artificial readout. Thus, we plotted the performance function in Figure 4C as a correlation coefficient between a binary variable, indicating whether a target combination of stimuli was present, and a continuous variable, indicating the ‘depolarization’ of the readout classifier (the so-called point-biserial coefficient of correlation). We also provide a formal proof (see Supp. Theorem 1) that, in this case, any point-biserial correlation coefficient that is significantly larger than zero indicates non-linear transformations attributable to neuronal processes.

The results are shown in Figure S21 for the analysis applied to the same dataset that was used in Figure 4C (red dashed line). In both cases, the classification performance was above chance level. As the inputs are assembled into a linear combination by the classifier, this result indicates that the non-linear transformations required for the XOR function classification must have been accomplished by the brain.

Interestingly, in one experiment (cat 3) the XOR classification performance was highest during the on-responses to the second stimulus (~250 ms), while in the other experiment (cat 2) peak performance was about 100 ms later, corresponding to the off-responses to the second stimulus. These results indicate that the computations accomplished in V1 (or before) are capable of supporting non-linear interactions between responses to successively presented stimuli and thus, that they can exploit some of the advantages of fading memory.

REFERENCES:

Minsky M, Papert SA (1969) **Perceptrons: An Introduction to Computational Geometry***.* Cambridge: MIT Press.

Natschläger T, Maass W (2005) Dynamics of information and emergent computation in

generic neural microcircuit models. Neural Networks 18(10): 1301-1308.

Schölkopf B, Smola AJ (2002) Learning with Kernels. Cambridge, MA: MIT Press. 644 p.

Vapnik VN (1998) Statistical Learning Theory. New York: Wiley. 736 p.

***Supplementary Theorem 1***

If the visual pathway maps time-dependent visual inputs linearly on the state of the cortical network, then, in combination with a static linear readout with a scalar output, these two pathways form also a linear transformation referred to as linear functional, where is a real-valued function of time, and is the set of vectors consisting of such functions of time. This linear functional maps the dimensional time dependent visual input on a real number representing the output of the static readout at a specific moment in time. For the following theorem, the visual input is composed of the sum of two visual input functions. The first (second) function represents the visual input during the first (second) time slot and is zero otherwise.

# Theorem: *Let* $s_{1+2}=s_{1}+s_{2}$ *denote the sum of two visual stimulus functions of time* $s_{1}\in\left\{s_{A},s_{C}\right\}$*and* $s_{2}\in\left\{s_{B},s_{D}\right\}$ *with* *and , then the correlation coefficient between the XOR functional defined by*

*and any non-constant linear functional*  *is zero*

**Proof:** The correlation coefficient between $f_{XOR}$ and $f_{L}$ is defined by

$$CC=\frac{\text{COV}\left(f_{XOR},f_{L}\right)^{2}}{\text{VAR}\left(f_{XOR}\right)\text{VAR}\left(f_{L}\right)}$$

where COV and VAR denote the covariance and the variance, respectively. Any linear functional  where $s_{1+2}=s_{1}+s_{2}$ denotes the sum of two functions of time $s_{1}\in\left\{s_{A},s_{C}\right\}$and $s_{2}\in\left\{s_{B},s_{D}\right\}$ with can be written as

$$f_{L}\left[s_{1}+s_{2}\right]=f_{L}\left[s_{1}\right]+f_{L}\left[s_{2}\right]$$

Furthermore

$$\begin{matrix} \text{COV}\left(f_{XOR},f_{L}\right)=\sum_{\left\{s_{1+2}\right\}} f_{XOR}\left[s_{1}+s_{2}\right]f_{L}\left[s_{1}+s_{2}\right]/4 \\ -\sum_{\left\{s_{1+2}\right\}} f_{XOR}\left[s_{1}+s_{2}\right]/4\cdot\sum_{\left\{s_{1+2}\right\}} f_{L}\left[s_{1}+s_{2}\right]/4 \\ =0\cdot(f_{L}\left[s_{A}\right]+f_{L}\left[s_{B}\right])/4 \\ +1\cdot(f_{L}\left[s_{A}\right]+f_{L}\left[s_{D}\right])/4 \\ +1\cdot(f_{L}\left[s_{C}\right]+f_{L}\left[s_{B}\right])/4 \\ +0\cdot(f_{L}\left[s_{C}\right]+f_{L}\left[s_{D}\right])/4 \\ -(f_{L}\left[s_{A}\right]+f_{L}\left[s_{B}\right]+f_{L}\left[s_{C}\right]+f_{L}\left[s_{D}\right])/4 \\ =0, \\ \end{matrix}$$

where $\left\{s_{1+2}\right\}$denotes $\left\{s_{A+B},s_{C+B},s_{A+D},s_{C+D}\right\}$***.*** The variance of non-constant $f_{L}$ is larger than 0 and thus $CC=0.$ ■
